# Aberrant expression of interleukin-23-regulated miRNAs in T cells from patients with ankylosing spondylitis

**DOI:** 10.1186/s13075-018-1754-1

**Published:** 2018-11-21

**Authors:** Ning-Sheng Lai, Hui-Chun Yu, Chien-Hsueh Tung, Kuang-Yung Huang, Hsien-Bin Huang, Ming-Chi Lu

**Affiliations:** 1 Division of Allergy, Immunology and Rheumatology, Dalin Tzu Chi Hospital, Buddhist Tzu Chi Medical Foundation, No. 2, Minsheng Road, Dalin Chiayi, 62247 Taiwan; 2 School of Medicine, Tzu Chi University, Hualien City, Taiwan; 3 Department of Life Science and Institute of Molecular Biology, National Chung Cheng University, Minxiong Chiayi, Taiwan

**Keywords:** Ankylosing spondylitis, IL-23, T cells, MicroRNAs, STAT3, Angiogenin

## Abstract

**Background:**

Interleukin (IL)-23 can facilitate the differentiation of IL-17-producing helper T cells (Th17). The IL-23/IL-17 axis is known to play a key role in the immunopathogenesis of ankylosing spondylitis (AS). We hypothesized that the expression of microRNAs (miRNAs, miRs) would be regulated by IL-23 and that these miRNAs could participate in the immunopathogenesis of AS.

**Methods:**

Expression profiles of human miRNAs in K562 cells, cultured in the presence or absence of IL-23 for 3 days, were analyzed by microarray. Potentially aberrantly expressed miRNAs were validated using T-cell samples from 24 patients with AS and 16 control subjects. Next-generation sequencing (NGS) was conducted to search for gene expression and biological functions regulated by specific miRNAs in the IL-23-mediated signaling pathway.

**Results:**

Initial analysis revealed that the expression levels of 12 miRNAs were significantly higher, whereas those of 4 miRNAs were significantly lower, in K562 cells after coculture with IL-23 for 3 days. Among these IL-23-regulated miRNAs, the expression levels of miR-29b-1-5p, miR-4449, miR-211-3p, miR-1914-3p, and miR-7114-5p were found to be higher in AS T cells. The transfection of miR-29b-1-5p mimic suppressed IL-23-mediated signal transducer and activator of transcription 3 (STAT3) phosphorylation in K562 cells. After NGS analysis and validation, we found that miR-29b-1-5p upregulated the expression of angiogenin, which was also upregulated in K562 cells after coculture with IL-23. Increased expression of miR-29b-1-5p or miR-211-3p could enhance interferon-γ expression.

**Conclusions:**

Among the miRNAs regulated by IL-23, expression levels of five miRNAs were increased in T cells from patients with AS. The transfection of miR-29b-1-5p mimic could inhibit the IL-23-mediated STAT3 phosphorylation and might play a role in negative feedback control in the immunopathogenesis of AS.

## Background

Ankylosing spondylitis (AS) is a chronic inflammatory disease characterized by axial skeletal involvement leading to spine deformities, increased disability, and mortality [1, 2]. The pathogenesis of AS is complex, and earlier research focused on the misfolding of human leukocyte antigen B27 [3], a major genetic risk factor in AS [4]. Recent studies revealed that the genetic variant of interleukin (IL)-23 receptor (IL-23R) was associated with the risk of AS [5]. Serum level of IL-23 is elevated in patients with AS compared with control subjects [6]. Other studies have also demonstrated that IL-23/IL-17-related signaling pathways could play a critical role in the pathogenesis of AS [7]. Most importantly, targeting IL-17 is a novel therapy for AS [8].

MicroRNAs (miRNAs, miRs) are short noncoding RNA molecules of 21–24 base pairs that control the expression of multiple gene targets at the post-transcriptional level. They play a crucial role in regulating both the innate and adaptive immune responses. One of our previous studies showed that dysregulated miRNAs in T cells from patients with AS could participate in the inflammatory response [9]. Many studies have also reported that the expression of miRNAs in whole blood, peripheral blood mononuclear cells (PBMCs), or serum from patients with AS could participate in bone erosion, cytokine expression, and autophagy in the pathogenesis of AS [10].

Few studies have addressed the IL-23/IL-17 axis-related miRNAs and their possible roles in the immunopathogenesis of AS [11]. We believe that many additional miRNAs regulated by IL-23 could be found to be aberrantly expressed in T cells from patients with AS, and these miRNAs could participate in the IL-23-related signaling pathway. In this study, we hypothesized that IL-23-regulated miRNAs in T cells from patients with AS could alter the expression of downstream target molecules and thereby contribute to the immunopathogenesis of AS.

## Methods

### Cell culture

Among the human myeloid and lymphoid cell lines, the mRNA expression of IL-23R is more abundant in K562 cells than in Jurkat cells [12]. We chose K562 cells for this study. K562 cells, a human erythroleukemia line, purchased from the American Type Culture Collection (Manassas, VA, USA) were cultured in medium with or without the presence of IL-23 (20 ng/ml; Sigma-Aldrich, St. Louis, MO, USA) for 3 days according to previous studies [13, 14] with some modifications. These cells were used for subsequent analysis.

### Isolation of T cells from patients and control subjects

A total of 24 patients fulfilling the Assessment of SpondyloArthritis international Society (ASAS) classification criteria [15] were recruited for this study. In addition, 16 healthy individuals were also recruited to serve as control subjects. Blood samples were collected just before taking oral medications and the administration of the next dose of biologic agent to minimize any effects of medications. All participants provided informed consent under a study protocol approved by the institutional review board of Dalin Tzu Chi Hospital, Buddhist Tzu Chi Medical Foundation (no. B10502002).

T cells were further purified by antihuman CD3-coated magnetic beads (IMag Cell Separation System; BD Biosciences, Franklin Lakes, NJ, USA), and the purities of T cells were all greater than 98% according to methods previously described [16].

### Measurement of ankylosing spondylitis disease activity

The Ankylosing Spondylitis Disease Activity Score (ASDAS) based on C-reactive protein (CRP) was used to evaluate disease activity in this study [17]. ASDAS-CRP was calculated using the following formula: 0.121 × back pain + 0.058 × duration of morning stiffness + 0.110 × patient global assessment + 0.073 × peripheral pain or swelling + 0.579 × ln (CRP + 1).

### RNA isolation for microarray and next-generation sequencing

Total RNA was extracted by using TRIzol® Reagent (Life Technologies, Carlsbad, CA, USA) according to the manufacturer’s instructions. The purified RNA was quantified at optical density 260 nm using an ND-1000 spectrophotometer (NanoDrop Technologies/Thermo Scientific, Wilmington, DE, USA), and quality was evaluated using a Bioanalyzer 2100 (Agilent Technologies, Santa Clara, CA, USA) with the RNA 6000 Nano Kit (Agilent Technologies).

### Microarray analysis of miRNAs

Total RNA (0.1 μg) was dephosphorylated and labeled with pCp-Cy3 by using the Agilent miRNA Complete Labeling and Hyb Kit (Agilent Technologies). Hybridization buffer (Agilent Technologies) was added to the labeled mixture to a final volume of 45 μl. The mixture was heated at 100 °C for 5 min and immediately cooled to 0 °C. Each 45-μl sample was hybridized onto an Agilent human miRNA Microarray R21 (Agilent Technologies) at 55 °C for 20 h. After hybridization, slides were washed in Gene Expression Wash Buffer at room temperature for 5 min and then in Gene Expression Wash Buffer 2 at 37 °C for 5 min (Agilent Technologies). Microarrays were scanned with an Agilent microarray scanner (model G2505C; Agilent Technologies) at 535 nm for Cy3. Feature Extraction software version 10.7.3.1 (Agilent Technologies) was used for image analysis. Microarray data were uploaded in the Gene Expression Omnibus (GEO) database of the National Center for Biotechnology Information [GEO:GSE118806].

### Measurement of expression of miRNAs

A real-time PCR-based method was used to quantify the expression levels of miRNAs following a protocol described previously [18]. Expression of the U6 small nuclear RNA was used as an endogenous control for data normalization.

### Measurement of expression of mRNAs

Expression levels of mRNA were quantified by real-time PCR using a one-step RT-PCR kit (TaKaRa, Shiga, Japan) on the ABI Prism 7500 Fast Real-Time PCR System (Applied Biosystems, Foster City, CA, USA). Conditions for the qPCR were 42 °C for 5 min and 95 °C for 10 s for RT, followed by 40 cycles of 95 °C for 5 s and 34 °C for 34 s. Expression of 18S ribosomal RNA was used as an endogenous control for data normalization.

### Western blot analysis

Cells were lysed with 1% NP-40 (Sigma-Aldrich) in the presence of a proteinase inhibitor and phosphatase inhibitor cocktail (Sigma-Aldrich). Seventy micrograms of the cell lysates were electrophoresed and transferred to a polyvinylidene difluoride sheet (Sigma-Aldrich). The membranes were blocked with 1% skim milk solution and then incubated with the primary antibodies for signal transducer and activator of transcription 3 (STAT3), phosphorylated STAT3 (Cell Signaling Technology, Danvers, MA, USA) and angiogenin (ANG) (Santa Cruz Biotechnology, Dallas, TX, USA), followed by horseradish peroxidase-conjugated secondary antibodies (Cell Signaling Technology). The cognate molecules were visualized using an enhanced chemiluminescence reaction (GE Healthcare Life Sciences, Marlborough, MA, USA).

### Transfection of miRNA

K562 cells (1 × 10^6^/ml) were electroporated with 1 μg of scrambled oligonucleotides or miRNA mimics (Ambion/Thermo Fisher Scientific, Austin, TX, USA) using the Gene Pulser MXcell electroporation system (Bio-Rad Laboratories, Hercules, CA, USA) using the condition described previously [18] and then cultured at 37 °C with a humidified atmosphere containing 5% CO_2_ for 24 h or 48 h for further analysis of miRNA expression or for Western blot analysis, respectively.

### Next-generation sequencing

We transfected K562 cells with miR-29b-1-5p mimic or scrambled oligonucleotides and then cultured them at 37 °C under a humidified atmosphere containing 5% CO_2_ for 48 h. The RNA was extracted according to the method described above. For the next-generation sequencing (NGS) analysis, all procedures were carried out according to the manufacturer’s protocol (Illumina, San Diego, CA, USA). In brief, library construction of all samples was performed by using the Agilent Technologies SureSelect Strand Specific RNA Library Preparation Kit for 75 single-end sequencing on the Solexa platform (Illumina). The sequence was directly determined using sequencing-by-synthesis technology via the TruSeq SBS Kit (Illumina). Raw sequences were obtained from the Illumina Pipeline software bcl2fastq v2.0 and expected to generate 30 million reads (or Gb) per sample. The sequencing procedure was performed by Welgene Biotech (Taipei, Taiwan). For the results analysis, the generated sequences went through a filtering process to obtain qualified reads initially. Trimmomatics (Illumina) was implemented to trim or remove the reads according to the quality score. Qualified reads after filtering low-quality data were analyzed using TopHat/Cufflinks [19] for gene expression estimation. The gene expression level was calculated as fragments per kilobase of transcript per million mapped reads. For differential expression analysis, CummeRbund was employed to perform statistical analyses of gene expression profiles. The reference genome and gene annotations were retrieved from the Ensembl database.

### Statistical analysis

Data are represented as the median and IQR or number (%) as appropriate. Simple and multiple linear regression analyses were used to calculate the correlation coefficients among different clinical parameters and expression levels of IL-23-regulated miRNAs in T cells of patients with AS. Statistical significance between patients with AS and control subjects was assessed using the Mann-Whitney *U* test. A *P* value < 0.05 was considered statistically significant. All analyses were performed with Stata software (StataCorp, College Station, TX, USA).

## Results

### Increased STAT3 phosphorylation in K562 cells after incubation with IL-23

**Fig. 1:**
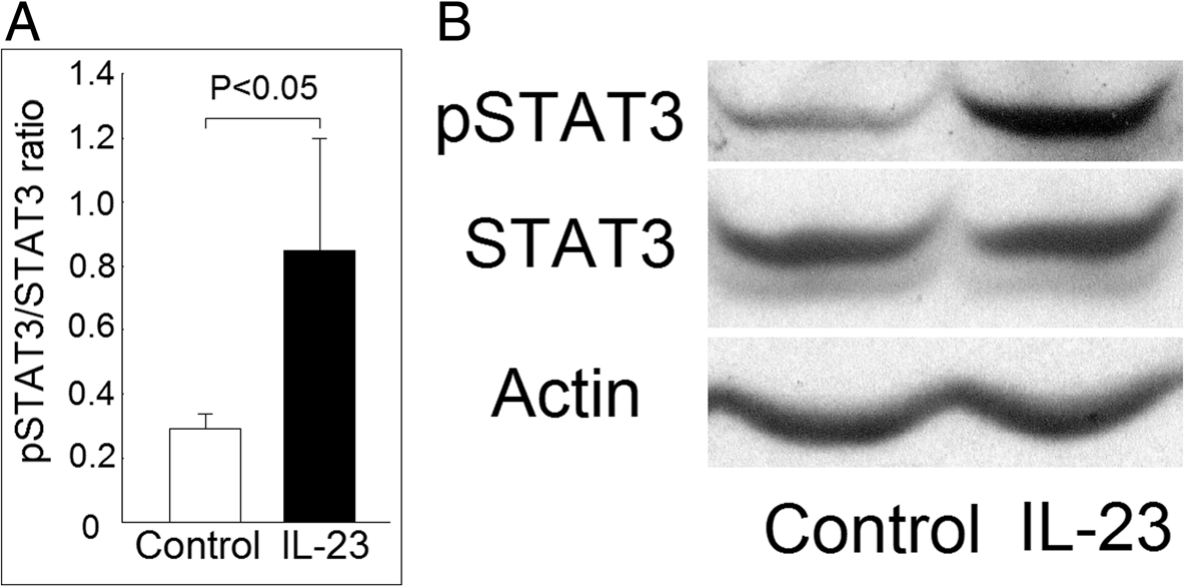
Effect of interleukin (IL)-23 on signal transducer and activator of transcription 3 (STAT3) protein phosphorylation in K562 cells. **a** The phosphorylation ratio of STAT3 increased in K562 cells after coculture with IL-23 (20 ng/ml) for 3 days compared with those cocultured with medium (control) only. **b** A representative case

First, we demonstrated that the phosphorylation ratio of STAT3, a key downstream signaling molecule of the IL-23 signaling pathway, was increased after coculture with IL-23 in K562 cells (Fig. 1a and b).

### Identification of the IL-23-regulated miRNA expression in K562 cells

**Fig. 2:**
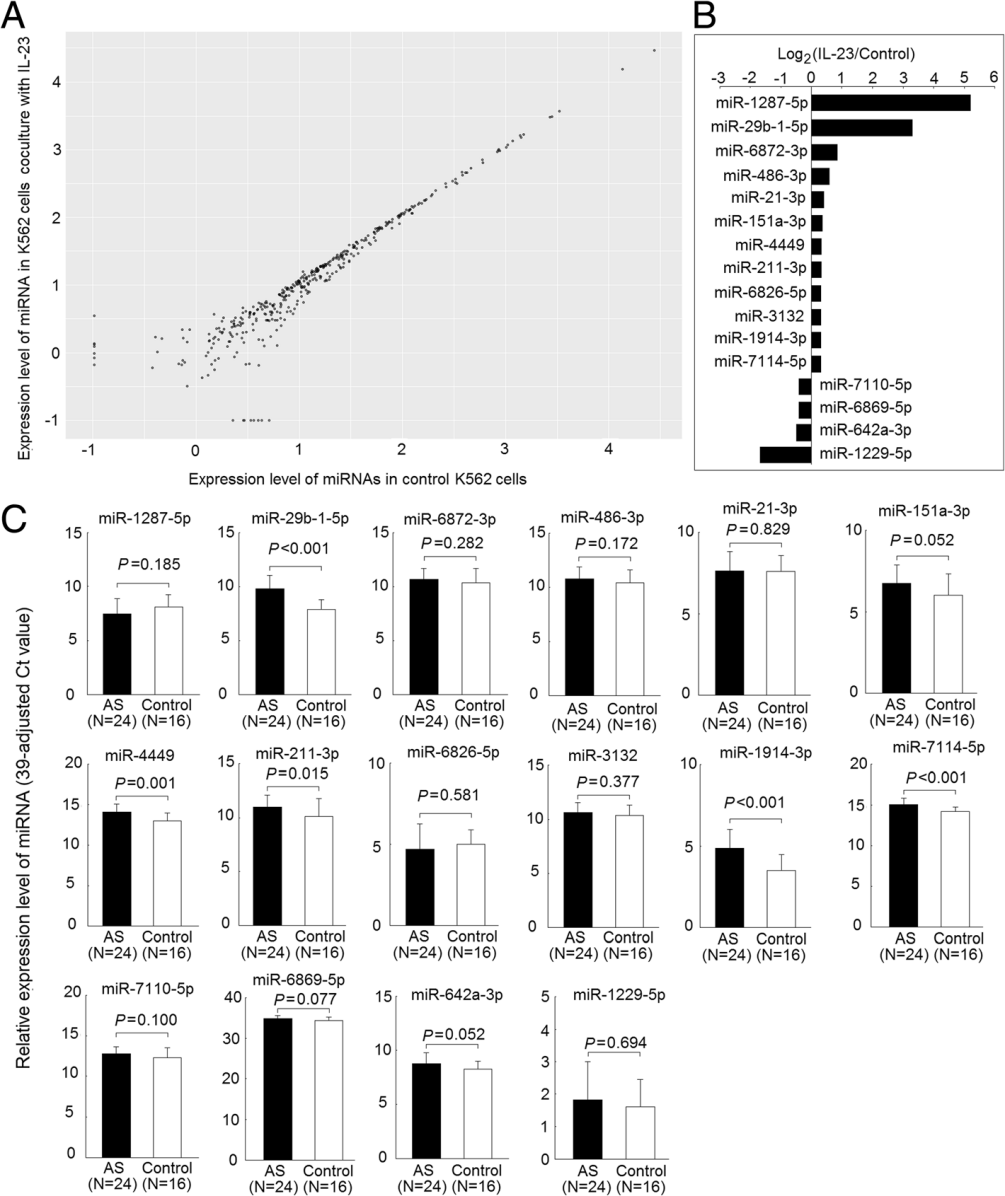
Altered expression of interleukin (IL)-23-regulated microRNAs (miRNAs) in T cells from patients with ankylosing spondylitis (AS) and from healthy control subjects. **a** Expression profiles of miRNAs in K562 cells cocultured with or without IL-23 (20 ng/ml) for 3 days, evaluated using microarray analysis. Each scatter spot represents the mean raw signals of miRNA in three repeats of each treatment. **b** The expression levels of 12 miRNAs were significantly higher, whereas the expression levels of 4 miRNAs were significantly lower, in K562 cells after coculture with IL-23 for 3 days (*P* < 0.05). **c** Increased expression of miR-29b-1-5p, miR-4449, miR-211-3p, miR-1914-3p, and miR-7114-5p in AS T-cell miRNA, compared with normal T cells after validation

Expression profiles of miRNAs in K562 cells cocultured with or without IL-23 (20 ng/ml) for 3 days are displayed in Fig. 2a, with each scatter spot representing the mean of three adjusted miRNA levels from each group. The expression levels of 12 miRNAs (miR-1287-5p, miR-29b-1-5p, miR-6872-3p, miR-486-3p, miR-21-3p, miR-151a-3p, miR-4449, miR-211-3p, miR-6826-5p, miR-3132, miR-1914-3p, and miR-7114-5p) were significantly higher, whereas the expression levels of 4 miRNAs (miR-7110-5p, miR-6869-5p, miR-642a-3p, and miR-1229-5p) were significantly lower, in K562 cells after coculture with IL-23 for 3 days (*P* < 0.05) (Fig. 2b).

### Expression profiles of IL-23-regulated miRNAs in T cells from patients with AS and control subjects

Expression levels of the IL-23-regulated miRNAs were investigated in T cells from patients with AS and control subjects. The demographic and clinical data of the 24 patients with AS and 16 control subjects are presented in Table 1. There were no differences in the distribution of age and sex between the two groups.

**Table 1:** Demographics and clinical data of patients with ankylosing spondylitis and healthy control subjects

	Patients with AS (*n* = 24)	Healthy control subjects (*n* = 16)	*P* value
Age (years)	39.5 (33.0–57.8)	39.0 (33.5–43.5)	0.313
Sex (M:F)	12:4	12:4	> 0.999
HLA-B27^+^	24 (100%)	–	
C-reactive protein (mg/dl)	0.24 (0.08–1.24)	–	
Medication			
NSAID	23 (95.8%)	–	
Salazopyrine	24 (100%)	–	
Anti-TNF therapy	14 (58.3%)	–	

*Abbreviations: AS* Ankylosing spondylitis, *HLA* Human leukocyte antigen, *–* Not determined, *NSAID* Nonsteroidal anti-inflammatory drugs, *TNF* Tumor necrosis factor

The expression of miR-29b-1-5p, miR-4449, miR-211-3p, miR-1914-3p, and miR-7114-5p (*P* < 0.05) was found to be higher in AS T cells than in controls (Fig. 2c). The fold changes of expression levels for these miRNAs compared with controls were as follows: miR-29b-1-5p, 3.71-fold, miR-4449, 2.13-fold, miR-211-3p, 1.85-fold; miR-1914-3p, 2.57-fold; and miR-7114-5p, 1.82-fold. After adjusting for age and sex, the expression levels of miR-29b-1-5p, miR-4449, miR-1914-3p, and miR-7114-5p miRNAs remained significantly higher in T cells from patients with AS than in those from control subjects.

### Correlations of miRNA expression levels and clinical parameters in patients with AS

The relationships between various clinical parameters and the expression levels of miRNAs in AS T cells were investigated using regression analyses (Table 2). With simple linear regression analysis, expression levels of miR-7114-5p showed a trend of correlation with the use of anti-tumor necrosis factor (anti-TNF) therapy (*P* = 0.099). After adjusting for age and sex using multiple linear regression analysis, patients with AS receiving anti-TNF therapy showed a significant 1.56-fold increment (*P* = 0.048; 95% CI, 1.00–2.43) in miR-7114-5p expression compared with those who did not receive anti-TNF therapy. Because we found that the fold changes of in miR-29b-1-5p expression level were the greatest among these IL-23-regulated miRNAs in T cells from patients with AS, we selected miR-29b-1-5p for subsequent analysis.

**Table 2:** Simple and multiple linear regression analyses for assessing the correlations among different clinical parameters and expression levels of IL-23-regulated miRNAs in T cells of patients with ankylosing spondylitis

	miR-7114-5p	miR-1914-3p	miR-211-3p	miR-4449	miR-29b-1-5p
Sex (M/F)	−0.112 (0.784)	0.317 (0.564)	0.473 (0.366)	0.294 (0.520)	−0.207 (0.731)
Age (per 10 years)	−0.085 (0.505)	0.021 (0.919)	0.030 (0.875)	−0.159 (0.337)	−0.031 (0.889)
CRP (per 1 mg/dl)	−0.005 (0.920)	0.008 (0.915)	0.073 (0.299)	0.054 (0.380)	0.020 (0.804)
Anti-TNF therapy (yes/no)	0.490 (0.099^a^)	0.351 (0.466)	0.330 (0.474)	0.286 (0.475)	0.029 (0.965)
ASDAS-CRP	−0.194 (0.250)	−0.941 (0.727)	−0.121 (0.639)	0.230 (0.304)	0.278 (0.358)

*Abbreviations: ASDAS* Ankylosing Spondylitis Disease Activity Score, *CRP* C-reactive protein, *TNF* Tumor necrosis factor

Values are correlation coefficients and (*P* values) from simple linear regression analyses

^a^After adjusting for age and sex in the multiple linear regression analysis, patients with AS using anti-TNF therapy had a significant 1.56-fold increase (*P* = 0.048; 95% CI, 1.00–2.43) in miR-7114-5p expression compared with those who did not receive anti-TNF therapy

### Decreased STAT3 phosphorylation in K562 cells after transfection of miR-29b-1-5p

**Fig. 3:**
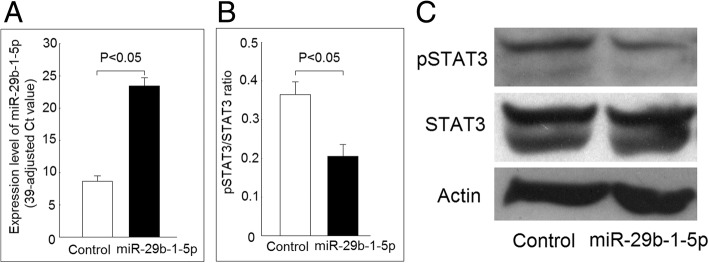
Effect of miR-29b-1-5p on signal transducer and activator of transcription 3 (STAT3) protein phosphorylation in K562 cells. **a** Increased miR-29b-1-5p expression in K562 cells after transfection with miR-29b-1-5p mimic versus scramble oligonucleotides. **b** The phosphorylation ratio of STAT3 decreased in K562 cells after transfection with miR-29b-1-5p mimic compared with those transfected with scramble oligonucleotides after culturing with medium for 48 h. **c** A representative case

We found that the expression levels of miR-29b-1-5p were significantly elevated in K562 cells 24 h after transfection with miR-29b-1-5p mimic compared with those transfected with scrambled oligonucleotides (as the control) (Fig. 3a). We found that the phosphorylation ratio of STAT3 decreased in K562 cells 48 h after transfection with miR-29b-1-5p mimic compared with the controls (Fig. 3b and c).

### Search of genes regulated by miR-29b-1-5p using next-generation sequencing

**Fig. 4:**
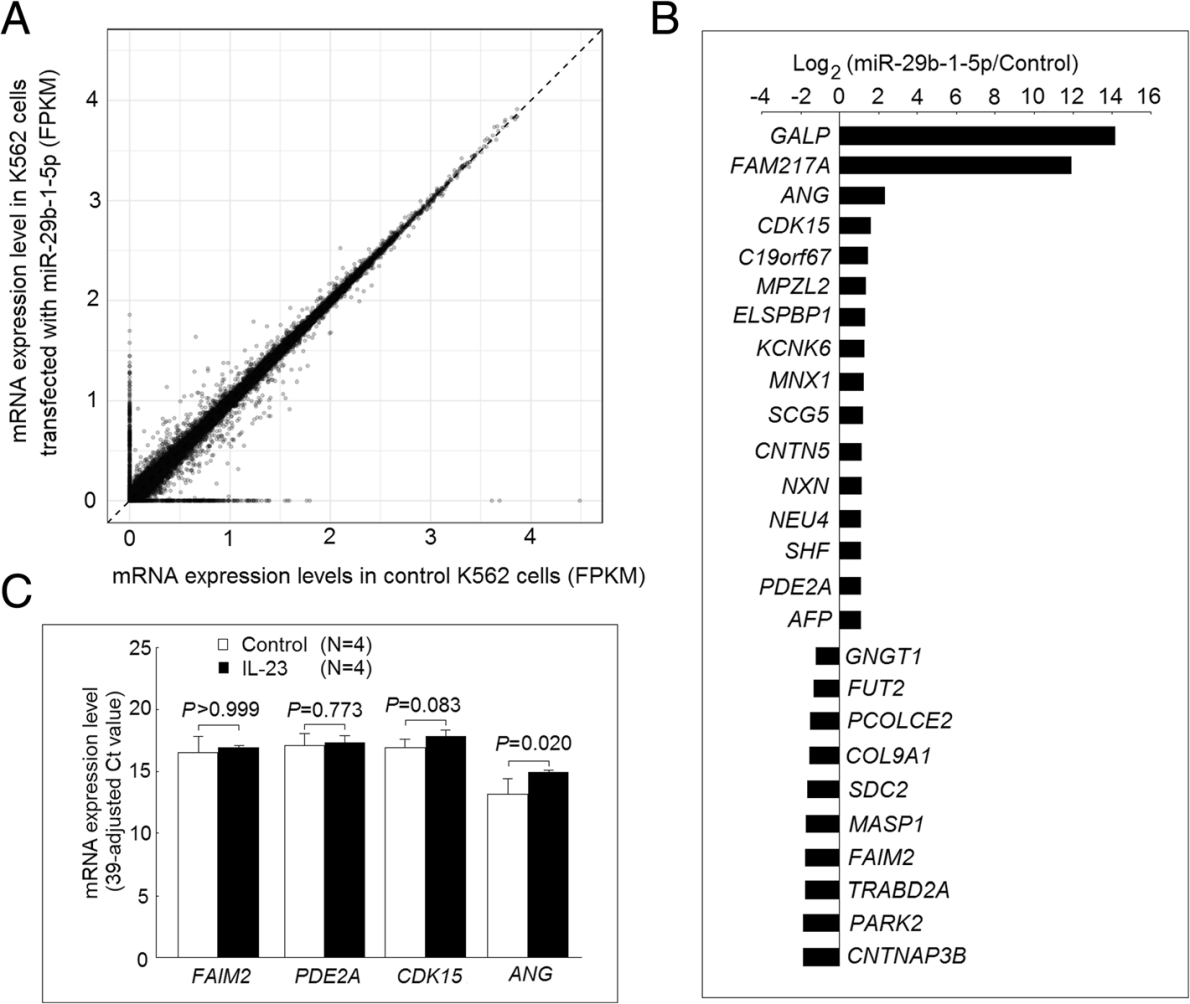
Identification of miR-29b-1-5p-regulated genes. **a** Expression profiles of messenger RNAs (mRNAs) in K562 cells transfected with miR-29b-1-5p mimic or scramble oligonucleotides then cultured with medium for 48 h were evaluated using RNA-Seq transcriptome analysis. Each scatter spot represents the mean raw signals of microRNA (miRNA) in three repeats of each treatment. **b** The RNA expression levels of 16 genes were significantly higher, whereas the RNA expression levels of 10 genes were significantly lower, in K562 cells after being transfected with miR-29b-1-5p mimic for 48 h. **c** Four genes potentially involved in the inflammatory responses were selected for further analysis. The mRNA expression levels of *ANG* were significantly elevated in K562 cells after coculture with interleukin (IL)-23 (20 ng/ml) for 3 days (Fig. 3c)

To search for the expression of genes regulated by miR-29b-1-5p, we performed gene expression analysis using NGS after transfecting K562 cells with miR-29b-1-5p mimic or scrambled oligonucleotides. We found that mRNA expression levels of 10 genes were decreased, whereas the expression levels of 16 genes were increased, in K562 cells after transfection with miR-29b-1-5p mimic compared with those transfected with scrambled oligonucleotides (*P* < 0.05) (Fig 4a and b).

### Involvement of miR-29b-1-5p-regulated genes in IL-23 signaling pathway

From among the previous identified miR-29b-1-5p regulated genes, we selected one downregulated gene, Fas apoptotic inhibitory molecule 2 (*FAIM2*), and three upregulated genes—phosphodiesterase 2A (*PDE2A*), cyclin-dependent kinase 15 (*CDK15*), and angiogenin (*ANG)*—that are potentially involved in the inflammatory responses for further analysis. The mRNA expression levels of *ANG*, but not *FAIM2*, *PDE2A*, or *CDK15*, were significantly elevated in K562 cells after coculture with IL-23 (20 ng/ml) for 3 days (Fig. 4c).

### Protein expression of angiogenin

**Fig. 5:**
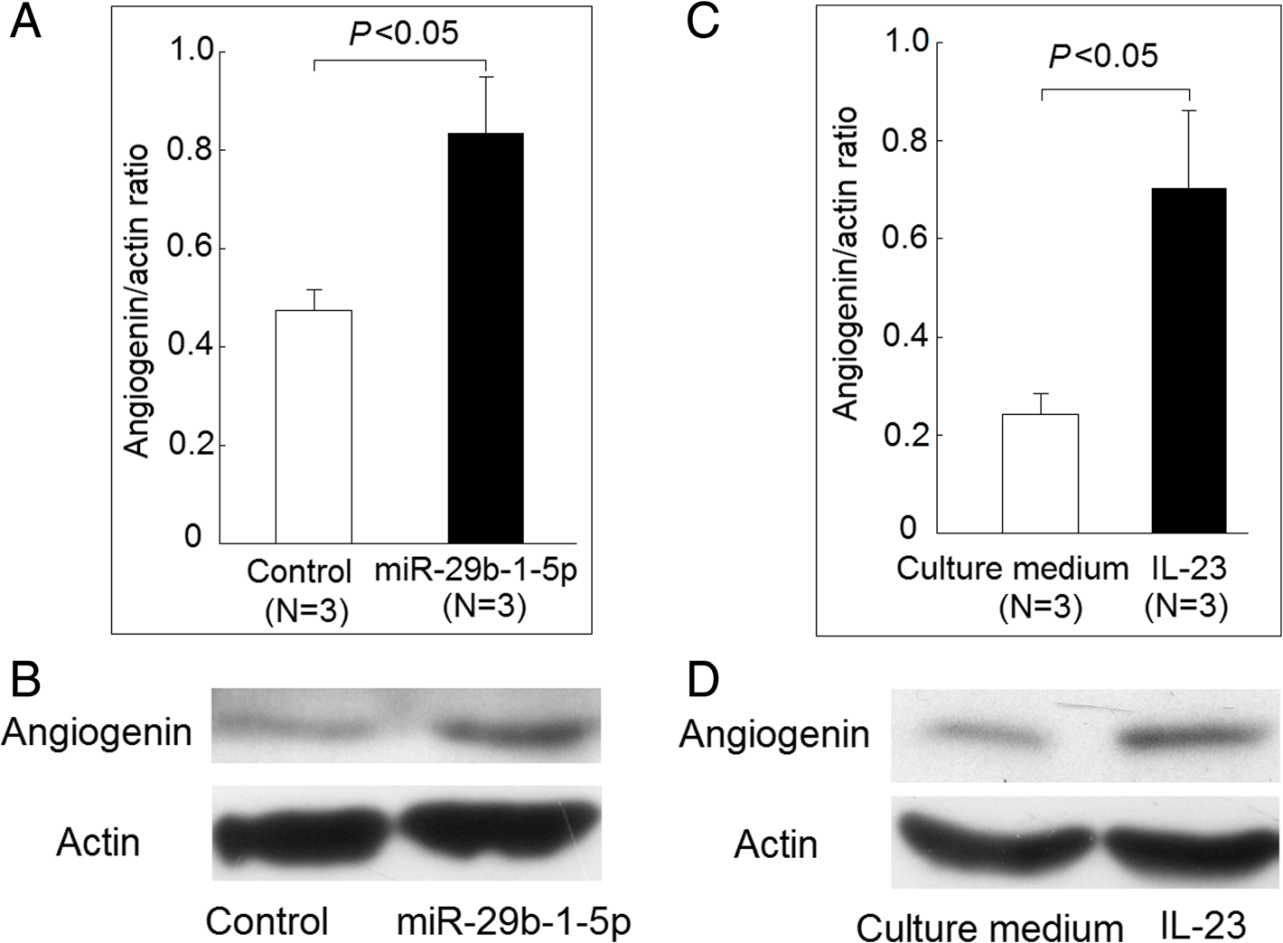
Protein expression of angiogenin was regulated by miR-29b-1-5p and interleukin (IL)-23. **a** The protein expression of angiogenin was elevated in K562 cells after being transfected with miR-29b-1-5p mimic for 48 h compared with those transfected with scramble oligonucleotides. **b** A representative case. **c** The protein expression of angiogenin was also increased in K562 cells after coculture with IL-23 for 3 days compared with those cultured with medium only. **d** A representative case

We confirmed that the protein expression of *ANG* was elevated in K562 cells after transfection with miR-29b-1-5p (Fig. 5a and b). The protein expression of *ANG* was also increased in K562 cells after coculture with IL-23 (Fig. 5c and d).

### Effect of miRNAs on expression levels of proinflammatory cytokines

**Fig. 6:**
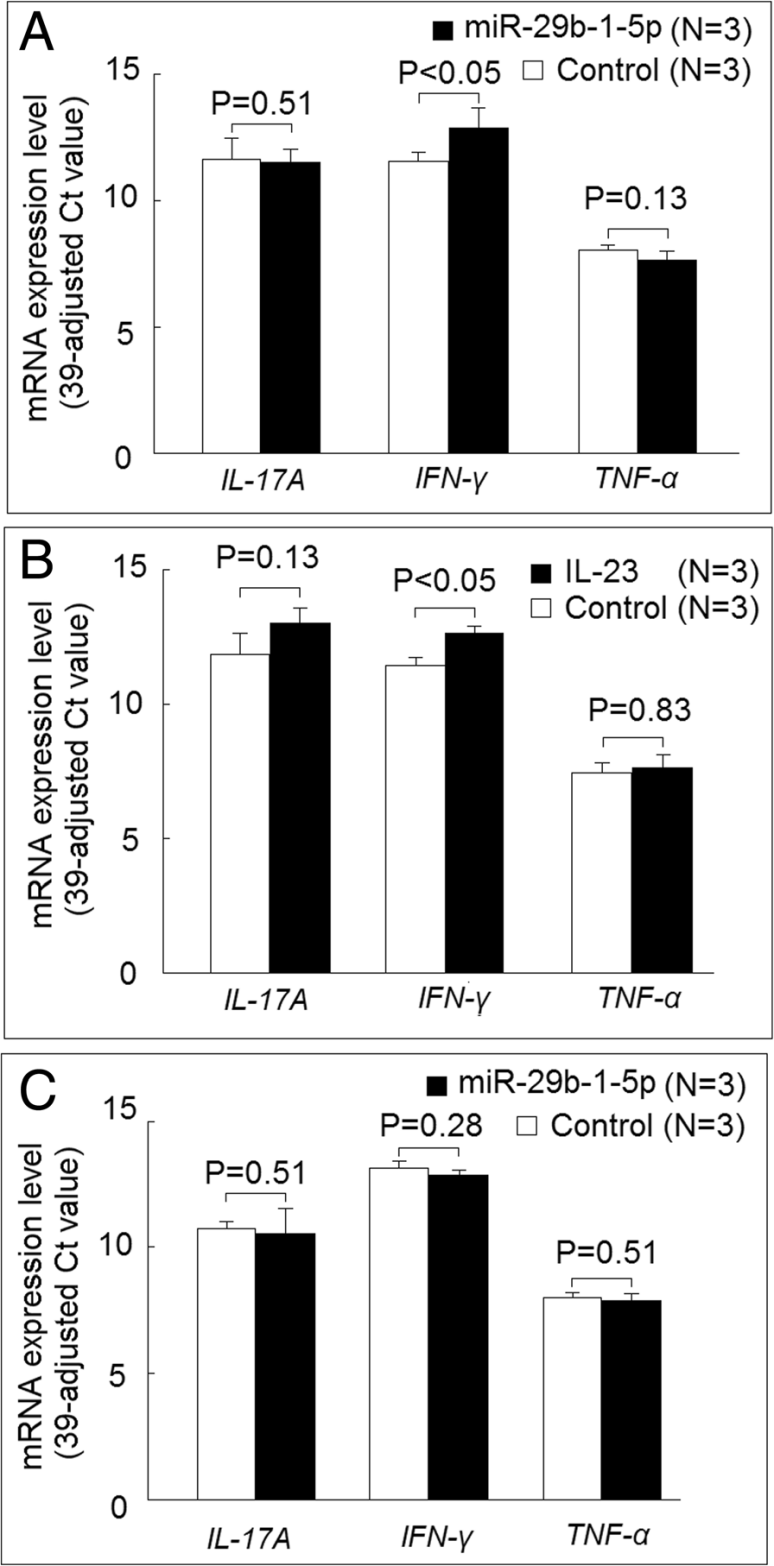
Effect of miR-29b-1-5p on proinflammatory cytokine expression. **a** The messenger RNA (mRNA) expression levels of interferon-γ (*IFN-γ*), but not interleukin (IL)-17A or tumor necrosis factor-α (*TNF-α*), were increased in K562 cells 24 h after transfection of miR-29b-1-5p mimic compared with those transfected with scrambled oligonucleotides. **b** The mRNA expression levels of *IFN-γ*, but not *IL-17A* or *TNF-α*, were increased in K562 cells after coculture with IL-23 (20 ng/ml) compared with those cultured with medium alone. **c** The K562 cells transfected with miR-29b-1-5p mimic or scrambled oligonucleotides were cultured with IL-23 (20 ng/ml) for 24 h, and there was no difference in the mRNA expression levels of *IFN-γ*, *IL-17A*, or *TNF-α* between these two groups

**Fig. 7:**
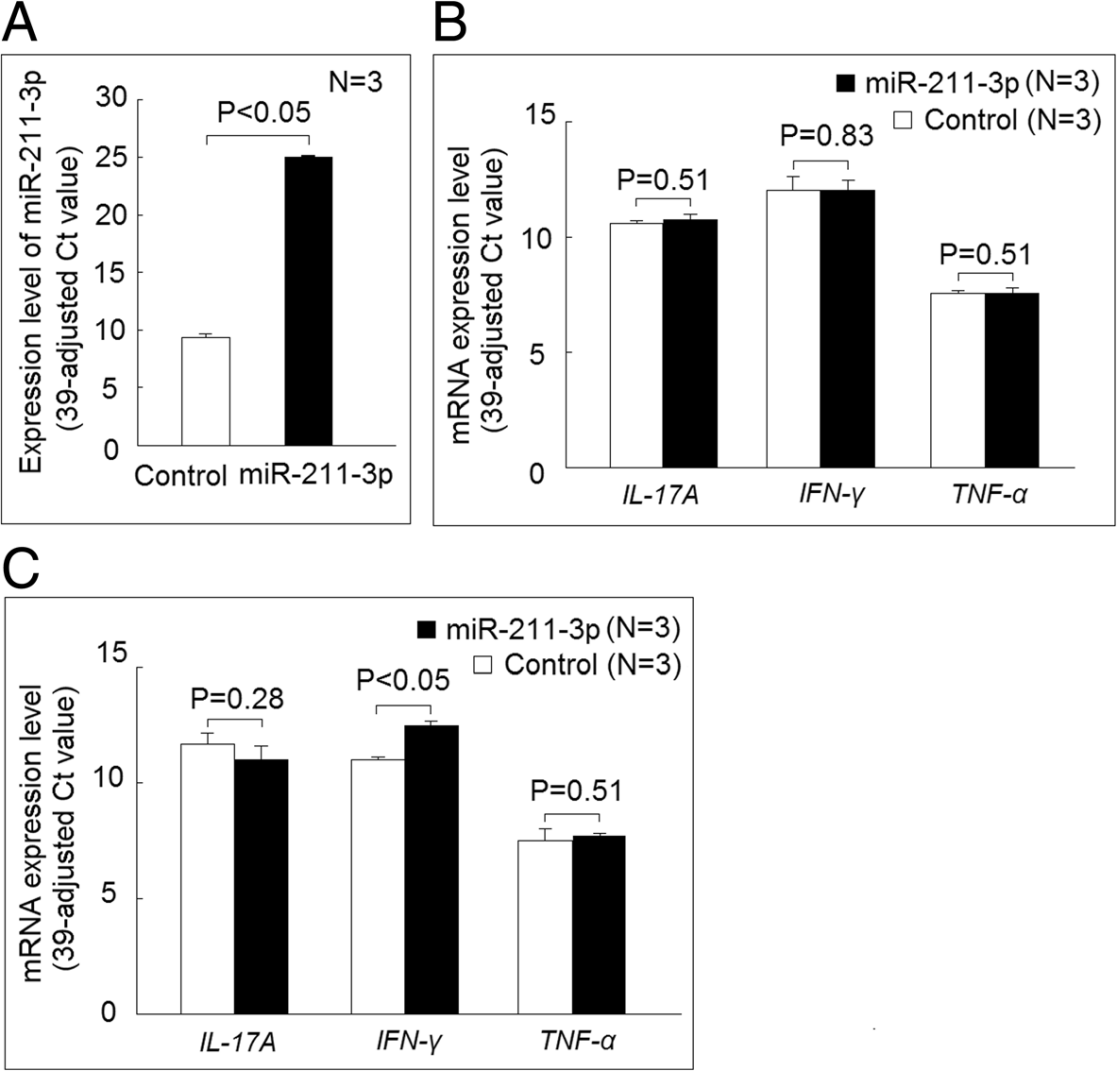
Effect of miR-211-3p in proinflammatory cytokine expression. **a** The expression levels of miR-211-3p dramatically increased after transfection with miR-211-3p mimic compared with those transfected with scrambled oligonucleotides (control). **b** There were no differences in the mRNA expression levels of interferon-γ (*IFN-γ*), interleukin (IL)-17A, or tumor necrosis factor-α (*TNF-α*) in K562 cells 24 h after transfection of miR-211-3p mimic compared with those transfected with scrambled oligonucleotides. **c** The K562 cells transfected with miR-211-3p mimic or scrambled oligonucleotides were cultured with IL-23 (20 ng/ml) for 24 h. The mRNA expression levels of *IFN-γ*, but not *IL-17A* or *TNF-α*, were increased in those transfected with miR-211-3p mimic compared with the control

We further surveyed the effect of increased expression of miR-29b-1-5p or miR-211-3p on the expression of proinflammatory cytokines. We found that increased miR-29b-1-5p expression could increase interferon-γ (*IFN-γ*) expression, but not *IL-17A* or *TNF-α* expression, in K562 cells cultured with medium alone (Fig. 6a). The addition of IL-23 could increase the expression of IFN-γ in K562 cells, but the transfection of miR-29b-1-5p did not affect the *IFN-γ*, *IL-17A*, or *TNF-α* expression in K562 cells cultured with IL-23 (Fig. 6b and c). We also confirmed that the expression levels of miR-211-3p were dramatically increased in K562 cells after transfection with miR-211-3p mimic (Fig. 7a). Increased expression of miR-211-3p did not affect the *IFN-γ*, *IL-17A*, or *TNF-α* expression in K562 cells cultured with medium alone (Fig. 7b). The transfection of miR-211-3p enhanced the mRNA expression of *IFN-γ*, but not of *IL-17A* or *TNF-α*, in K562 cells cocultured with IL-23 (Fig. 7c).

## Discussion

IL-23, a member of the IL-12 family, is a heterodimeric cytokine secreted by several types of immune cells, such as natural killer cells and dendritic cells [20]. Patients with AS have elevated serum IL-23 levels compared with control subjects [6]. In addition to AS, IL-23 is also involved in the pathogenesis of several other autoimmune diseases, such as inflammatory bowel disease and psoriasis, which all belong to the category of spondyloarthritis [21]. However, few studies have addressed the possible effect of IL-23 on the regulation of miRNA expression [22–24]. In this study, K562 cells instead of Jurkat cells were used as a platform for screening the IL-23-regulated miRNA expression because Jurkat cells had low expression levels of IL-23 receptor compared with K562 cells [12]. The differential expression levels of IL-23-regulated miRNAs were validated in T-cell samples from patients with AS and control subjects.

Although several studies have attempted to identify the potential roles of miRNAs in the pathogenesis of AS using PBMCs, serum, plasma, or whole blood [10], few studies have addressed the role of miRNAs in T-cell dysfunction of AS [6, 9–11, 25]. In this study, we found that the expression levels of five miRNAs (miR-29b-1-5p, miR-4449, miR-211-3p, miR-1914-3p, and miR-7114-5p) were higher in T cells from patients with AS. One of our earlier studies showed that three miRNAs were overexpressed in AS T cells [9]. In that study, only the expression levels of 270 miRNAs were explored. However, in the present study, we used a microarray that contained 2549 miRNA expression profiles. Therefore, a large number of miRNAs were expected to be found differentially expressed in AS T cells. In addition, miR-4449 is overexpressed in multiple myeloma [26], and it is interesting that the abnormal expression of IL-23 plays a role in the pathogenesis of multiple myeloma [27]. The role of other miRNAs deserves further study.

For the functional aspect of IL-23-regulated miRNAs, we found that the expression of miR-7114-5p was elevated in T cells from patients with AS. Anti-TNF therapy was associated with increased expression levels of miR-7114-5p. Milanez et al. found that the use of anti-TNF therapy in patients with AS did not affect the plasma level of IL-23 [28]. The use of anti-TNF therapy might indicate that these patients with AS had more severe or more active disease. We found that miR-29b-1-5p could suppress the phosphorylation of STAT3, a critical downstream transcription factor of the IL-23 signaling pathway that is required for Th17 cell differentiation [29]. Therefore, the increased expression of miR-29b-1-5p after exposure to IL-23 could be a negative feedback signal for the IL-23 signaling pathway. We found that increased *ANG* expression in K562 cells after transfection with miR-29b-1-5p mimic. We also confirmed that the addition of IL-23 or increased expression of miR-29b-1-5p could upregulate the protein expression of *ANG*. *ANG*, also known as ribonuclease 5, was initially known to induce new blood vessel formation. More recently, many biological functions, such as regulating cell proliferation, survival, migration, invasion, and/or differential are shown to be regulated by *ANG* [30, 31]. In inflammatory responses, ANG could inhibit the TANK-binding protein kinase 1-mediated nuclear factor-κB translocation, and this could suppress inflammatory responses [32]. Because IL-17 could enhance *ANG* expression in fibrocytes, it might play a role in the IL-23/IL-17-related signaling pathway [33]. Eleftheriadis et al. showed that ANG could inhibit T-cell apoptosis [34], and the biological function of *ANG* in T cells needs to be further explored.

## Conclusions

The expression levels of miR-29b-1-5p, miR-4449, miR-211-3p, miR-1914-3p, and miR-7114-5p were shown to be higher in AS T cells among the IL-23-regulated miRNAs. Increased expression of miR-29b-1-5p could suppress IL-23-mediated STAT3 phosphorylation and increase *ANG* expression in the IL-23 signaling pathway.

## Abbreviations

ANG: Angiogenin
AS: Ankylosing spondylitis
ASAS: Assessment of SpondyloArthritis international Society
ASDAS: Ankylosing Spondylitis Disease Activity Score
CDK15: Cyclin-dependent kinase 15
CRP: C-reactive protein
FAIM2: Fas apoptotic inhibitory molecule 2
HLA: Human leukocyte antigen
IFN-γ: Interferon-γ
IL: Interleukin
miRNAs: MicroRNAs
NSAID: Nonsteroidal anti-inflammatory drug
PBMC: Peripheral blood mononuclear cell
PDE2A: Phosphodiesterase 2A
STAT: Signal transducer and activator of transcription
TNF-α: Tumor necrosis factor-α
